# Effect of different silica coatings on the toxicity of upconversion nanoparticles on RAW 264.7 macrophage cells

**DOI:** 10.3762/bjnano.12.3

**Published:** 2021-01-08

**Authors:** Cynthia Kembuan, Helena Oliveira, Christina Graf

**Affiliations:** 1 Institut für Chemie und Biochemie, Physikalische und Theoretische Chemie, Freie Universität Berlin, Takustraße 3, D-14195 Berlin, Germany; 2 Department of Biology & CESAM, University of Aveiro, 3810-193 Aveiro, Portugal; 3 Hochschule Darmstadt - University of Applied Sciences, Fachbereich Chemie- und Biotechnologie, Stephanstr. 7, D-64295 Darmstadt, Germany

**Keywords:** cytotoxicity, ion release, RAW 264.7 macrophage cell line, silica coating, upconversion nanoparticles

## Abstract

Upconversion nanoparticles (UCNPs), consisting of NaYF_4_ doped with 18% Yb and 2% Er, were coated with microporous silica shells with thickness values of 7 ± 2 and 21 ± 3 nm. Subsequently, the negatively charged particles were functionalized with *N*-(6-aminohexyl)-3-aminopropyltrimethoxysilane (AHAPS), which provide a positive charge to the nanoparticle surface. Inductively coupled plasma optical emission spectrometry (ICP-OES) measurements revealed that, over the course of 24h, particles with thicker shells release fewer lanthanide ions than particles with thinner shells. However, even a 21 ± 3 nm thick silica layer does not entirely block the disintegration process of the UCNPs. 3-(4,5-dimethylthiazol-2-yl)-2,5-diphenyltetrazolium bromide (MTT) assays and cell cytometry measurements performed on macrophages (RAW 264.7 cells) indicate that cells treated with amino-functionalized particles with a thicker silica shell have a higher viability than those incubated with UCNPs with a thinner silica shell, even if more particles with a thicker shell are taken up. This effect is less significant for negatively charged particles. Cell cycle analyses with amino-functionalized particles also confirm that thicker silica shells reduce cytotoxicity. Thus, growing silica shells to a sufficient thickness is a simple approach to minimize the cytotoxicity of UCNPs.

## Introduction

Upconversion nanoparticles (UCNPs) convert excitation radiation with long wavelengths to a short-wavelength emission. Since biological molecules do not have an upconversion mechanism, imaging with UCNPs avoids autofluorescence. Besides, UCNPs have further advantages for applications in life science, such as deep penetration depth, minimal photodamage, and high resistance to photobleaching [1–9]. Moreover, high thermal and photochemical stability, as well as high chemical inertness and relatively low toxicity are also claimed advantages [7,10–11]. Due to these unique features, UCNPs have already been used in medical and biological applications, such as multimodal bioimaging, drug delivery, photodynamic therapy, and biosensing [9,12–17].

However, UCNPs in aqueous dispersions undergo minor disintegration, which also results in the quenching of their luminescence intensity [8,10,18–23]. This concentration-dependent effect is especially significant when the dispersions are highly diluted [8,19,22], when the pH value is low [22], or when ions forming lanthanide salts with a low solubility (such as phosphates) are present [10,20,24], which is relevant for their application in physiological solutions. The cytotoxicity of F^−^ ions is in the range of a few millimoles per liter [25–26]. The release of F^−^ ions can induce oxidative stress and cause apoptosis. In addition, intracellular redox homeostasis and gene expression can be modulated [26]. Lanthanide ions are usually not reported as highly toxic. However, they can interact with proteins, enzymes, and other biomolecules [27–28] and might also cause oxidative damage or lipid peroxidation [29].

When UCNPs are applied in life sciences, it is usually necessary to modify their surfaces with hydrophilic ligands or layers [21,30–31]. These coatings can also prevent, to some extent, the interaction between UCNPs and the aqueous environment and, consequently, reduce their disintegration processes. Several authors have reported the use of protective coatings, such as poly(acrylic acid) and poly(allylamine hydrochloride) [18], multichelating phosphonate [30,32–33], block copolymers [34], amphiphilic polymers [8,21], or polysulfonates [22]. Silica shells can also be used to protect UCNPs surfaces from dissolution. In contrast to a more complex polymeric coating, silica surfaces can be easily functionalized with a wide range of coupling agents and biomolecules, and the interior of the silica shell can be modified by integrating dye molecules, for example. However, amorphous silica is a porous material. A typical Stöber silica has a pore size of around 1–4 nm [35–36]; therefore, a thin silica coating shell cannot completely inhibit the dissolution of UCNPs [37]. The thickness of silica shells on UCNPs can be easily adjusted over a wide range [38]. Lathinen et al. have shown that even a thin silica coating shell of <2 nm or of 5 nm can already reduce the luminescence quenching of UCNPs in an aqueous dispersion [19]. Besides, several studies revealed that silica-coated UCNPs have a low toxicity in vitro and in vivo compared with other nanoparticles [7,11,39]. Amorphous silica is highly stable over a broad pH range. Thus, it is expected that it protects UCNP cores [40] even if the pH is reduced to values of approx. 4.5–5 in lysosomes during cellular uptake processes [41].

In the present work, the cytotoxicity of UCNP cores coated with silica shells was investigated in the macrophage cell line RAW 264.7. RAW 264.7 cells are particularly sensitive to the treatment with nanoparticles [42–44]. They are an established model of activated macrophages and they actively take up nanomaterials from biological media. This way, RAW264.7 cells mimic the behavior of macrophages and other immune cells, which eliminate foreign substances from the organism. Moreover, they have already been applied in studies involving uncoated NaGdF_4_ [42] and silica particles [43–46].

Upconversion cores consisting of NaYF_4_ doped with 18% Yb and 2% Er were synthesized. Microporous silica shells with two different thickness values were grown onto these cores to enable the investigation of a possible relation between the degree of cytotoxicity, particle size, and silica shell thickness. The particles were subsequently functionalized with *N*-(6-aminohexyl)-3-aminopropyltrimethoxysilane (AHAPS), which provides the nanoparticle surface with a positive charge, increasing their interaction with the cell membrane. The particles were characterized by scanning transmission electron microscopy (STEM), dynamic light scattering (DLS), electrophoretic light scattering (ELS), and inductively coupled plasma optical emission spectrometry (ICP-OES). Before the cell experiments, the stability of the particles in cell culture media was investigated via DLS and ELS. The cytotoxicity of the UCNPs was determined by MTT assays and cell cycle analysis. The UCNP uptake potential was evaluated by flow cytometry through the measurement of side-scattered light, which is proportional to changes in cell granularity or to the internal complexity.

## Results and Discussion

### Preparation and characterization of upconversion nanoparticles

**Figure 1:**
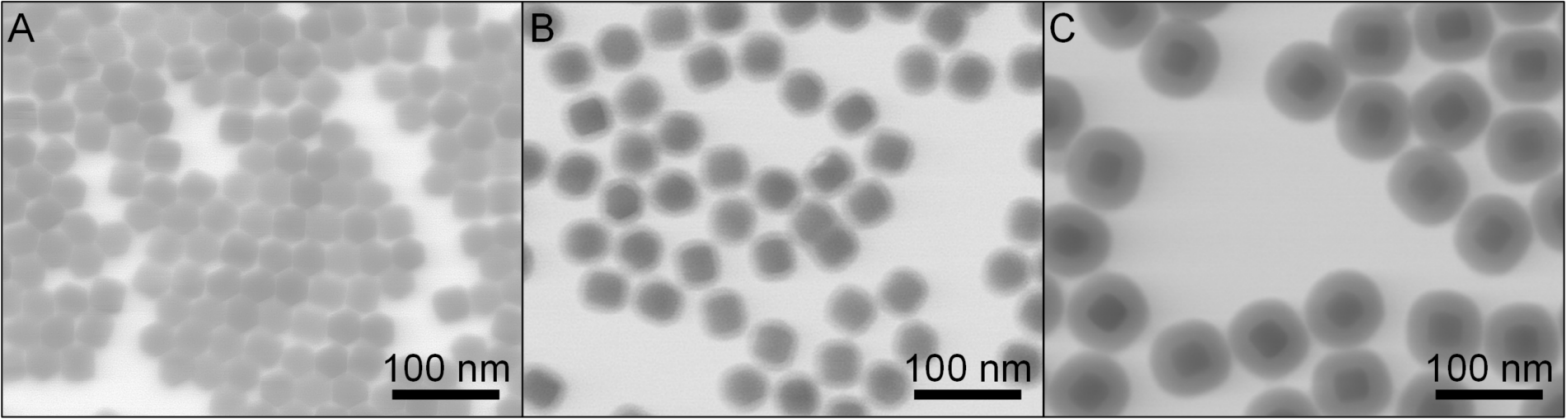
STEM image of (A) oleate-coated UCNPs (NaYF_4_: 18% Yb, 2% Er). (diameter = 33 ± 2 nm), (B) UC@thin (thickness of the silica shell (*t*_SiO2_) = 7 ± 2 nm); (C) UC@thick (*t*_SiO2_ = 21 ± 3 nm).

Oleate-functionalized NaYF_4_:Yb,Er nanocrystals were prepared by a thermal decomposition method [47], yielding spherical particles with a low polydispersity (Figure 1A, STEM diameter (*d*_STEM_) = 33 ± 2 nm). The hydrodynamic diameter (*Z*-average) was 47 ± 1 nm (polydispersity index, PDI = 0.38 ± 0.05). ICP-OES measurements yielded a percentual molar ratio of Y/Yb/Er = [74 ± 1]:[25 ± 1]:[2 ± 0.5]. The XRD diffractogram shows a predominantly hexagonal crystal structure (JCPDS No. 00-028-1192), which is typical for such UCNPs (Supporting Information File 1, Figure S1) [47]. The core was coated with two different silica layers: 7 ± 1 nm for the thin-shelled silica layer and 21 ± 2 nm for the thick-shelled silica layer (samples UC@thin, Figure 1B, and UC@thick, Figure 1C, respectively). A thicker silica shell protects the UCNP core more efficiently than a thinner silica shell by reducing the diffusion of water molecules to the UCNPs and also by reducing a possible leakage of ions from the core. The UC emission spectrum shows the typical green and red Er^3+^ emission bands of Er- and Yb-doped NaYF_4_ NP (Supporting Information File 1, Figure S2) [48–50]. The shape of the UC luminescence spectra is not influenced by the thickness of the silica coating, as reported in our previous work [38]. Additionally, UC@thin and UC@thick samples were surface-functionalized with *N*-(6-aminohexyl)-3-aminopropyltrimethoxysilane (AHAPS) (samples UC@thin_NH_2_ and UC@thick_NH_2_). AHAPS was chosen as a surface ligand due to its ability to provide the particles with a positive surface charge [51]. Positively charged silica particles interact more efficiently with the negatively charged cell membrane than negatively charged particles [45], which can also cause an enhanced uptake [51–52]. This process is supported by the fact that the hydrodynamic diameter of the AHAPS-functionalized particles is small enough for the particles to be internalized via the endocytic uptake [51]. (3-Aminopropyl)trimethoxysilane (APS) was not chosen as the amine ligand due to the increased aggregation of APS-functionalized particles in the cell culture medium [51].

In addition, particles with a modified silica shell were prepared such that the coupling product of rhodamine B isothiocyanate and APS (RBITC-APS) was coupled into the silica shell. Samples with two different thickness values were prepared: 9 ± 2 nm for the thin-shelled samples (sample UC@thin_RBITC_NH_2_) and 22 ± 2 nm for the thick-shelled samples (sample UC@thick_RBITC_NH_2_). The silica shells of the dye-doped samples were slightly thicker than those of the samples without the dye, as APS and RBITC-APS slightly increase the porosity of the silica shell. Consequently, identical amounts of silica per particle result in slightly thicker shells. As a reference system, pure silica nanoparticles with a size of 50 nm were also coupled with RBITC and functionalized with AHAPS (sample SiO_2_ @RBITC_NH_2_). STEM images of each sample are shown in Supporting Information File 1, Figure S3. The STEM data of all the particles is summarized in Table 1.

**Table 1:** STEM diameter, silica shell thickness as well as hydrodynamic diameter (*Z*-average, *Z*-ave), polydispersity index, and zeta potential (ZP) values of silica-coated UCNPs in EtOH, water, and in supplemented DMEM complete medium.

Samples	*d*_STEM_ [nm]	*t*_SiO2_ [nm]	EtOH				Water				DMEM	
				
			*Z*-ave [nm]	ZP [mV]	PDI		*Z*-ave [nm]	ZP [mV]	PDI		*Z*-ave [nm]	PDI

UC@thin_NH_2_	48 ± 2	8 ± 2	105 ± 1	34 ± 1	0.099 ± 0.005		128 ± 5	26 ± 2	0.118 ± 0.004		318 ± 13	0.720 ± 0.045
UC@thick_NH_2_	75 ± 2	21 ± 2	145 ± 1	37 ± 2	0.177 ± 0.015		295 ± 2	29 ± 1	0.258 ± 0.028		220 ± 2	0.460 ± 0.010
UC@thin_RBITC_NH_2_	50 ± 2	9 ± 2	127 ± 1	30 ± 2	0.117 ± 0.014		138 ± 2	26 ± 1	0.172 ± 0.028		97 ± 8	0.575 ± 0.098
UC@thick_RBITC_NH_2_	76 ± 3	22 ± 2	118 ± 1	27 ± 2	0.065 ± 0.009		139 ± 2	19 ± 1	0.161 ± 0.023		144 ± 2	0.367 ± 0.049
UC@thin	47 ± 2	7 ± 2	80 ± 2	24 ± 1	0.112 ± 0.004		104 ± 1	31 ± 2	0.203 ± 0.006		93 ± 1	0.460 ± 0.004
UC@thick	75 ± 3	21 ± 3	98 ± 2	21 ± 1	0.037 ± 0.006		142 ± 1	29 ± 1	0.098 ± 0.014		125 ± 3	0.159 ± 0.011
SiO_2_@RBITC_NH_2_	52 ± 3	—	98 ± 1	16 ± 1	0.100 ± 0.010		103 ± 2	10 ± 1	0.100 ± 0.010		208 ± 5	—

The dispersion behavior and changes in the surface charge of the samples in various media (ethanol, water, and Dulbecco's Modified Eagle Medium (DMEM) supplemented with 10% fetal bovine serum (FBS), 1% glutamine, 1% fungizone, and 1% penicillin) were studied by conducting DLS and ELS measurements. The DLS and ZP results are also shown in Table 1.

The zeta potential changed from negative to positive after AHAPS functionalization due to the positive surface charge of the amine group in the AHAPS ligand. The zeta potential values of the AHAPS-functionalized samples slightly decreased after transfer from ethanol to water, as reported in several publications [51–52], and, consequently, their hydrodynamic diameter values increased. The zeta potential of the non-functionalized particles is more negative in water than in ethanol, and, in this case, the *Z*-average value also increases.

The *Z*-average values of the samples after redispersion in DMEM were lower than in water, except for the samples UC@thin_NH_2_, UC@thick_RBITC_NH_2_, and SiO_2_@RBITC_NH_2_. The lower *Z*-average values of these samples may indicate an increased stabilization by a protein corona [52–56]. However, the high ionic strength of the cell culture medium (*I* = 168 mmol/L) reduces the electrostatic stabilization. Besides, the proteins in the DMEM cell culture medium supplemented with 10% FBS contribute to the measured hydrodynamic diameter values [51]. FBS consists mostly of bovine serum albumin. The *Z*-average value of the supplemented DMEM used in this study (without particles) was 13 ± 1 nm, and the corresponding PDI was 0.380 ± 0.003. This causes an additional reduction of the hydrodynamic diameter compared to water, which also explains the large PDI of the samples.

Izak-Nau et al. investigated the aggregation of silica nanoparticles that occurred after redispersion in buffered solution and in physiological medium [54]. They reported that various proteins in a medium containing FBS were adsorbed onto the surface of bare SiO_2_ and amine-functionalized SiO_2_ nanoparticles, forming a protein corona with a new surface charge, which depended on the type of proteins that built the corona. The adsorbed protein corona, consisting of the proteins present in FBS, could increase or reduce the stability of the particles and, consequently, their hydrodynamic diameter [53–57]. The non-functionalized samples, which have a negative surface charge due to the presence of silanol groups on the surface, were more stable in the cell culture medium than the amino-functionalized particles, which is in line with previous findings [51,54]. The adsorption of a protein corona makes the surface charge of the nanoparticles more negative; hence, it reduces the stability of positively charged particles [52,58–59]. Although the particles in this work showed increased aggregation in DMEM, they can still be taken up by macrophages, such as RAW 264.7 cells [45]. This is also indicated by the different cytotoxicity degrees of the samples in RAW 264.7 cells (the cytotoxicity of the samples was dose-dependent) and by the flow cytometry results (see below).

### Ion release experiments

For the investigation of released lanthanide ions, UC@thin_NH_2_ and UC@thick_NH_2_, as representative samples of thin- and thick-shelled samples, were redispersed in water.

For a better comparison with the results obtained from cell culture experiments (see below), samples with 200 μg/mL of silica-coated UCNPs or with 200 μg/mL of uncoated UCNP cores were prepared, allowed to stand for 24 h, and centrifuged with centrifuge tubes containing a filter unit (pore size: 3000 NWCO) to separate the UCNPs from possibly released ions. A concentration of 200 μg/mL was chosen, since this was the highest concentration used in the cytotoxicity experiments. Hence, the concentration of released ions is representative of the maximum concentration of released ions, which should correlate with the cytotoxicity results. The filtrates were measured by ICP-OES regarding their content of Y^3+^, Yb^3+^, and Er^3+^. Additionally, a certain amount of the three corresponding lanthanide chlorides was dissolved in water to yield lanthanide ion concentration values of 1.0 ± 0.1 and 2.0 ± 0.1 ppm. These solutions were, then, centrifuged with centrifuge tubes containing a filter unit. The filtrate was measured via ICP-OES to determine the percentage of ions filtered through the centrifuge tube filter.

Similar preliminary tests were also performed with UCNPs and lanthanide chlorides in DMEM. However, only Er^3+^ could be detected, with a high measurement uncertainty, in the filtrate of lanthanide chloride solutions. Lanthanide ions are known to bind to phosphate in phosphate-buffered saline (PBS) and form stable lanthanide phosphates [20]. Since DMEM contains Na_2_HPO_4_, it can be assumed that the lanthanide ions will also bind to these mentioned compounds. Therefore, a quantitative analysis of ion release was not performed in DMEM.

Table 2 shows the percentages of filtered ions detected by ICP-OES after 24 h of redispersion in water. Supporting Information File 1, Table S1 shows the amounts of detected filtered ions, from initial ion concentration values of 1.0 ± 0.1 and 2.0 ± 0.1 ppm, after dissolution in water. The recovery rate of the ions in water was the lowest for Er^3+^ (approx. 6%), followed by Yb^3+^ (17–21%) and Y^3+^ (38–45%). Due to these results and to the relatively low Er^3+^ content of the samples, data regarding Er^3+^ ions was not further considered.

**Table 2:** Percentages of released lanthanide ions from silica-coated UCNPs obtained via ICP-OES after 24 h of dispersion in water.

Sample	*c* = 200 μg/mL UCNP cores			*c* = 200 μg/mL silica-coated UCNPs		
		
	Y [%]	Yb [%]		*c* (UCNP cores) [µg/mL]	Y [%]	Yb [%]

UC@thin_NH_2_	0.97 ± 0.01	0.18 ± 0.03		96	1.88 ± 0.07	0.41 ± 0.05
UC@thick_NH_2_	0.33 ± 0.02	0.20 ± 0.02		33	0.97 ± 0.03	0.15 ± 0.08

The sample UC@thin_NH_2_ showed a significantly higher percentage of released lanthanide ions after 24 h of redispersion in water and centrifugation when compared to UC@thick_NH_2_ (Table 2). However, the difference would have been much larger if only the reduction of the diffusion rate through the three times thicker shell had delayed the dissolution of the UCNPs [60]. The percentage values for Y^3+^ release are generally higher than that for Yb^3+^. This can be partially explained by the lower content of Yb^3+^ and the lower recovery rate of Yb^3+^ compared to Y^3+^, which decreases further with decreasing concentration. Nonetheless, the difference is more significant than what is expected from these considerations. Lahtinen et al. have also observed that, in comparison to Yb^3+^, a significantly higher molar fraction of Y^3+^ is released from NaYF_4_:Yb,Er nanocrystals [19]. Dong et al. reported an analogous observation for the ratio of Y^3+^ and Gd^3+^ during a partial disintegration of NaGdF_4_:Y^3+^,Tb^3+^ [61]. This finding can be explained assuming that Y^3+^ ions are concentrated at the nanoparticle surface and, consequently, are more easily dissolved compared to the other ions [19].

The samples with 200 μg/mL silica-coated UCNPs show a higher percentage of released ions compared to the samples with 200 μg/mL UCNP cores, since the dissolution of NaYF_4_:Yb,Er UCNPs in water is limited by its low solubility product [62–63]. The ICP-OES data shows that the release of lanthanides from UCNPs even with a silica coating is not negligible; however, a thicker layer reduces the release of lanthanides. Lahtinen et al. reported that NaYF_4_:Yb,Er particles with a similar diameter (26–31 nm) but with a poly(acrylic acid) coating release more than 7% of their F^−^ ions in 24 h at a concentration of 50 µg/mL [19]. This comparison suggests that a 7 nm thick silica layer is enough to significantly reduce the disintegration process. In line with our findings, Saleh et al. also observed, in a study published during the review process of this work, by measuring released F^−^ and Y^3+^ ions that the dissolution of UCNPs in water can be almost completely suppressed by a thick (73 nm) microporous silica shell. In the case of a 10 nm thick silica shell, they observed that the amount of released ions increases with time [57].

### Cytotoxicity studies

**Figure 2:**
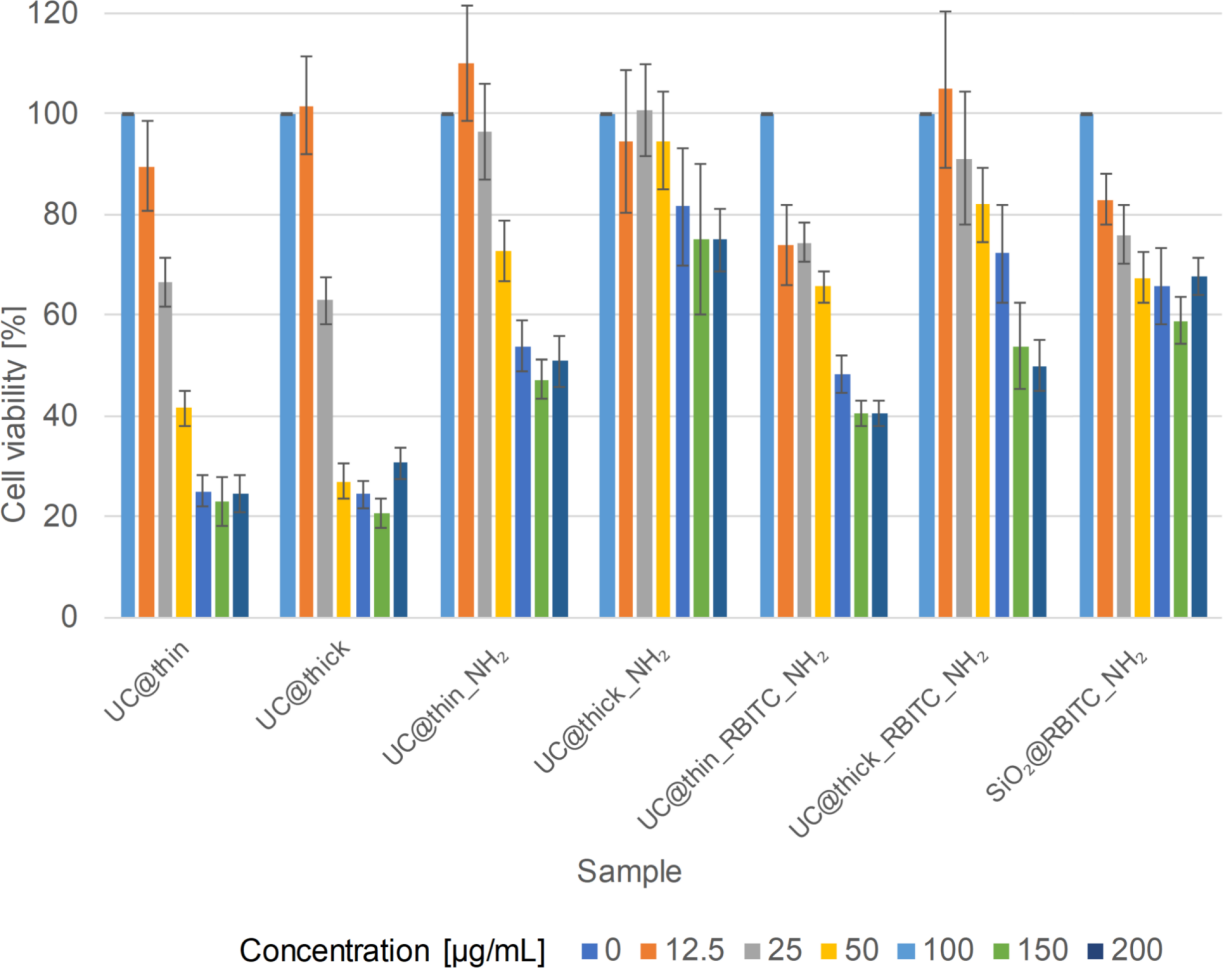
MTT assay results of silica-coated UCNPs and SiO_2_ nanoparticles on RAW 264.7 cells.

Figure 2 shows the viability results for the RAW 264.7 cells upon exposure to UCNPs. Due to the results of the ion release experiments, the same total particle mass values were compared. The cytotoxicity of UC@thin_NH_2_ was higher than that of UC@thick_NH_2_ in RAW 264.7 cells. At the highest particle concentration (*c* = 200 µg/mL), the cell viability after exposure to UC@thin_NH_2_ was approx. 51 ± 5%, whereas in the UC@thick_NH_2_ sample, the cell viability was 75 ± 6%. At the lowest concentration (*c* = 12.5 µg/mL) the cell viability was 110 ± 12% for UC@thin_NH_2_ and 95 ± 14% for UC@thick_NH_2_. UC@thin_RBITC_NH_2_ caused a slightly higher cytotoxicity than UC@thin_NH_2_, especially at lower concentration values, such as 12.5 and 25 µg/mL. At these concentration values, the cytotoxicity of the former sample was approx. 74 ± 1%. In general, UC@thick_NH_2_ was the least cytotoxic particle type for all samples. At the highest concentration values (*c* = 150 and 200 µg/mL) of UC@thick_NH_2_, no significant difference in the cell viability was observed between the two values. The cytotoxicity of pure silica without a UCNP core (sample SiO_2_@RBITC_NH_2_) was also measured. The cell viability at the lowest concentration was 83 ± 5%, and at the highest concentration was 68 ± 4%, indicating a moderate cytotoxicity.

The aggregation state of the nanoparticles can also influence the formation of their protein corona and, consequently, the release of ions in the cells. However, no indication of such effects were found in the present study. The sample UC@thin_NH_2_ has a larger hydrodynamic diameter than the sample UC@thick_NH_2_ in DMEM and the situation is reversed for sample UC@thin_RBITC_NH_2_ and sample UC@thick_RBITC_NH_2_. However, in both cases the cell viability increases with shell thickness. Since the light scattering is proportional to the 6th power of the particle size, small changes in the aggregation state of the nanoparticles cause significant changes in the *Z*-average in DLS.

Both non-functionalized samples were more cytotoxic compared to the amino-functionalized particles. UC@thin exhibits only a slightly higher degree of cytotoxicity than UC@thick. Nabeshi et al. investigated the cytotoxicity of non-modified, amine-functionalized, and carboxyl-functionalized 70 nm SiO_2_ NPs in RAW 264.7 cells [46]. They observed that unmodified SiO_2_ nanoparticles had the highest cytotoxicity due to the higher degree of uptake into cells. In contrast, the amine-functionalized particles were only adsorbed onto the cell membrane. Similar results were also obtained by Kurtz-Chalot et al., in which SiO_2_ nanoparticles with a high positive charge were more adsorbed than taken up by cells compared to the corresponding non-modified particles [45]. Xia et al. investigated cell type-dependent cytotoxicity in RAW 264.7, epithelial (BEAS-2B), human microvascular endothelial (HMEC), hepatoma (HEPA-1), and pheochromocytoma (PC-12) cells after exposure to amine-functionalized polystyrene nanoparticles (NH_2_-PS) [64]. They observed that lysosomal permeabilization and mitochondrial damage happened in RAW 264.7 cells but not in the other cell types. The particles were cytotoxic to RAW 264.7 and BEAS-2B cells but not to other cells. The nanoparticles perturbed the proton pump activity in RAW 264.7 cells, causing osmotic swelling and lysosomal rupture.

According to these results, RAW 264.7 cells internalize negatively charged particles to a greater extent than positively functionalized ones, causing the former to have higher cytotoxicity on RAW 264.7 cells, as it was also observed in this work. Nevertheless, the thicker silica shell reduces the degree of cytotoxicity of the amino-functionalized samples in macrophages more than that of non-functionalized particles. Possibly, the ions released at the cell membrane also can reduce cell viability.

In this work, the cell viability of silica-coated particles is higher than that of non-coated NaGdF_4_ nanoparticles. Wysokińska et al. investigated such particles, with average sizes between 4 and 249 nm and IC_50_ values below 2 µg/mL, via 3-(4,5-dimethylthiazol-2-yl)-5-(3-carboxymethoxyphenyl)-2-(4-sulfophenyl)-2*H*-tetrazolium (MTS) assays [42].

Despite the significant effects of the silica shell on cell viability, it should be noted that other factors, besides shell and surface functionalization, can influence the cytotoxicity of lanthanide-containing particles and might be entangled with the present observations. Liu et al. recently observed that the cellular concentration of Eu- and Bi-doped GdVO_4_ nanoparticles in polymer shells decreases with incubation time due to the occurrence of proliferation and exocytosis [65]. Such effects can be related to the functionalization and size of the nanoparticles as well as to ion release, and modulate the toxicity of lanthanide-containing particles as a function of time.

The obtained data shows that a particle concentration of up to 12.5 μg/mL does not lead to a critical decline in cell viability for all the particles under study. This quantity can be used as a reference point for the biomedical implementation of these particles. In addition, an incubation time longer than 24 h can be used in future experiments to determine the influence of prolonged contact with UCNP-containing particles and a small amount of released ions on cytotoxicity.

### Cellular uptake

Flow cytometry can provide qualitative and quantitative information about internalized particles in cells or particles adsorbed onto cellular membranes, relying on the fact that when cells internalize nanoparticles they increase their internal complexity [66–67]. Several publications have shown that side-scattering correlates with the concentration of nanoparticles attached to or taken up by cells [67–73].

**Figure 3:**
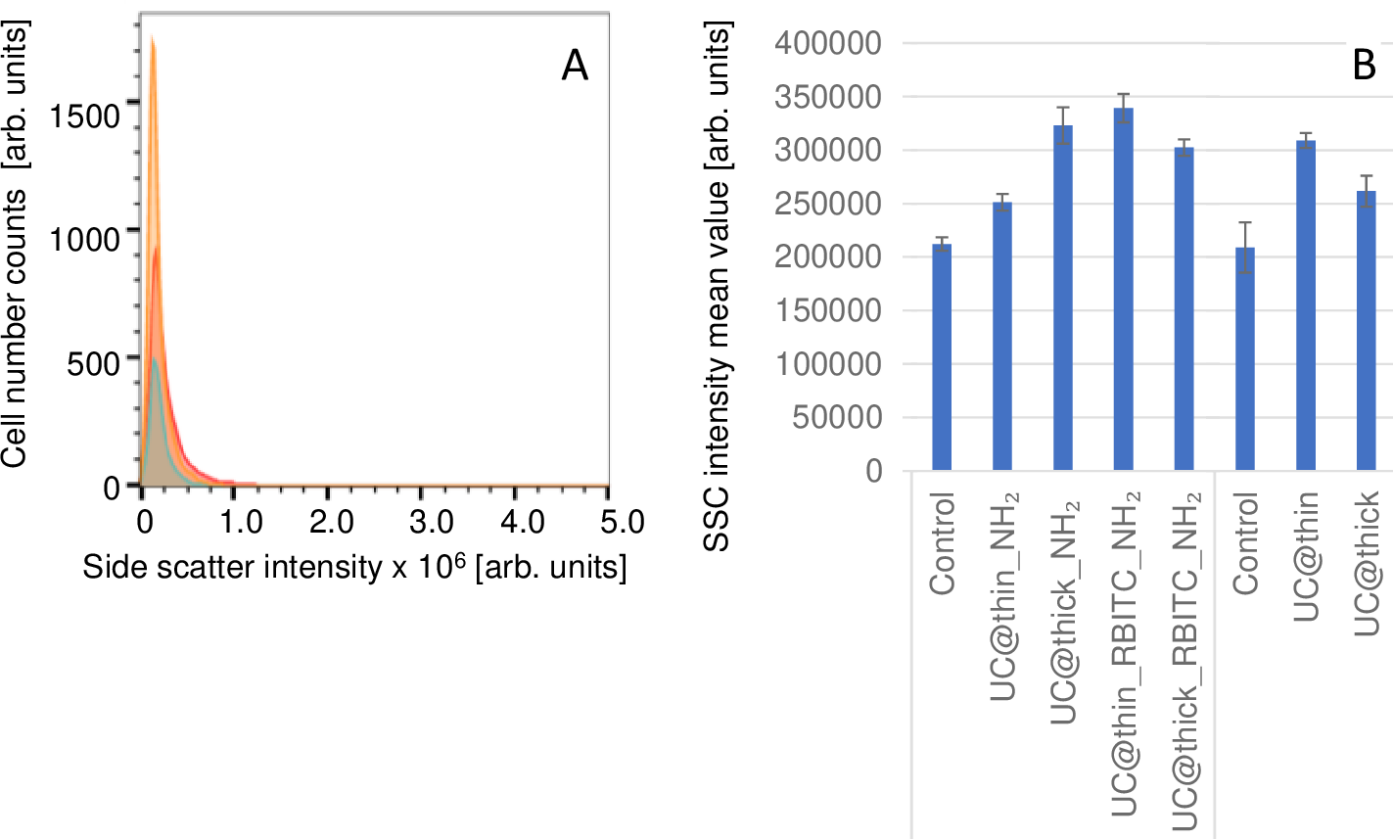
(A) SSC histograms of RAW 264.7 cells after particle exposure for 24 h at 37 °C. UC@thin_NH_2_ is marked by a blue-framed peak, UC@thick_NH_2_ is marked by a red-framed peak, and the control is marked by a yellow-framed peak. (B) Summary of mean SSC flow cytometry measurements on all samples in RAW 264.7 cells after particle exposure for 24 h at 37 °C.

The flow cytometry measurements were carried out in cells incubated with nanoparticles (*c* = 100 µg/mL) for 24 h at 37 °C. Figure 3 shows side-scatter (SSC) histograms of RAW 264.7 cells after exposure to UC@thin_NH_2_ (blue-framed area) and UC@thick_NH_2_ (red-framed area). The data for non-particle-treated controls is marked as a yellow-framed area. After 24 h, the SSC mean value for UC@thin_NH_2_ was (251 ± 8) × 10^3^, and that of UC@thick_NH_2_ was (323 ± 17) × 10^3^, while the control value was (212 ± 6) × 10^3^. The percentage increase of the SSC mean value for UC@thin_NH_2_ was 18 ± 5%, whereas for UC@thick_NH_2_ it was 52 ± 9%, indicating a higher increase of cell granularity and a higher uptake rate for UC@thick_NH_2_ compared to the samples with thin-shelled particles. The MTT cytotoxicity assay showed a higher cytotoxicity for UC@thin_NH_2_ compared to UC@thick_NH_2_, meaning that a higher increase in the side-scattering signal of the thick-coated particles does not correspond to a higher cytotoxicity. Therefore, although more thick-shelled particles were taken up, they are less toxic to the cells than a smaller quantity of thin-shelled particles.

Figure 3 shows a bar chart of the SSC mean values for RAW 264.7 cells. The flow cytometry measurements of UC@thin and UC@thick were performed at a different time than the rest of the samples. Hence, each had their own negative control samples.

In the case of UC@thin_RBITC_NH_2_ and UC@thin samples, the particles with thinner shells had higher SSC mean values than those with thicker shells, indicating greater changes in cellular granularity after exposure to the nanoparticles (i.e., a higher amount of incorporated particles). This was not the case for the sample UC@thin_NH_2_, which had the lowest SSC intensity of all coated UCNP samples. However, this does not go along with a higher cytotoxicity since the thin-coated samples had a higher degree of cytotoxicity in the MTT assay in comparison to the thick-coated particles (Figure 2). The cytotoxicity of the thin-coated samples must have been caused by other effects that did not result in a stronger increase in cell granularity, such as a higher release of ions and a related reduction in cell viability, as indicated by the ion-release experiments and MTT data (Figure 2 and Figure 3, respectively).

### Cell cycle analysis

**Figure 4:**
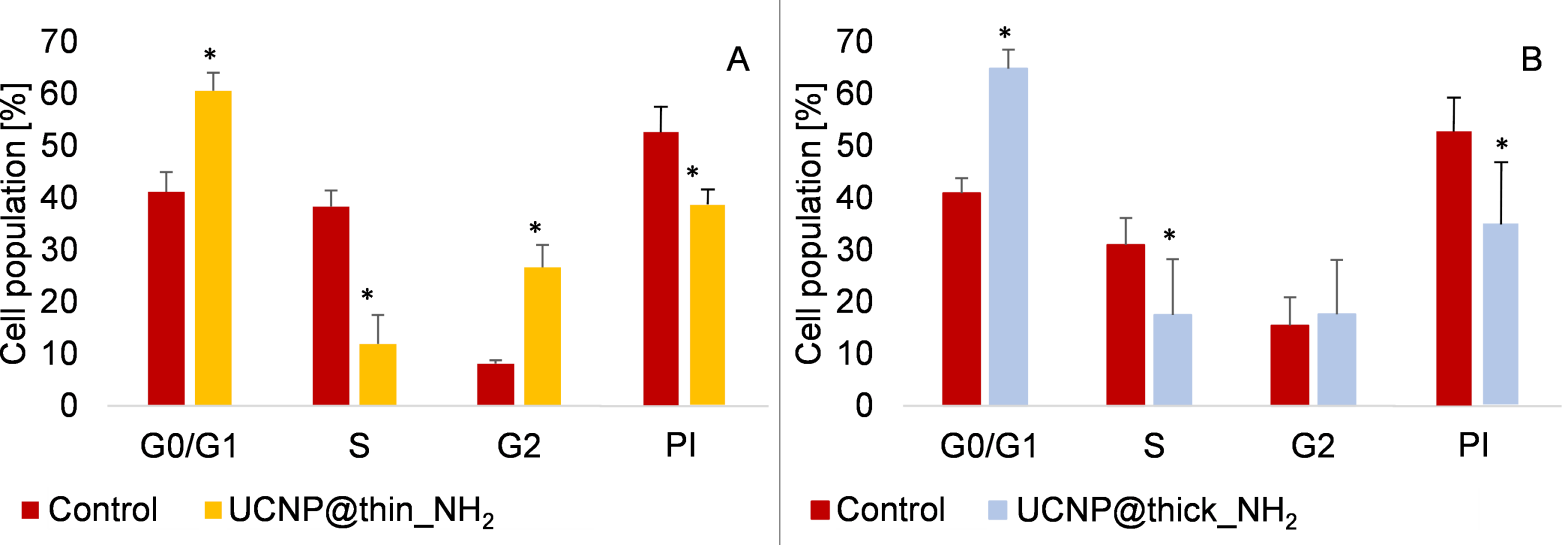
Effect of (A) UC@thin_NH_2_ (*t*_SiO2_ = 8 ± 2 nm) and (B) UC@thick_NH_2_ (*t*_SiO2_ = 21 ± 2 nm) on the cell cycle dynamics of RAW 264.7 macrophages after 24 h of exposure. The concentration used was 200 µg/mL. The asterisk indicates significant differences relative to the control *p* < 0.05.

To gain a deeper understanding of the effect of silica-coated UCNPs on RAW 264.7 macrophages, an analysis of the cell cycle dynamics of UCNP@thin_NH_2_ and UCNP@thick_NH_2_ samples was carried out. The cell cycle consists of four parts: The rest phase (G0); the first gap phase (G1), in which the cells grow and produce enzymes necessary for cell division; the synthesis phase (S), in which the DNA is replicated; and the second gap phase (G2), in which the cell continues to grow further and to perform processes that are necessary for mitosis [74]. Both silica-coated samples show a significant increase in the G0/G1 phase compared to control cells (not treated with nanoparticles) (Figure 4). Accordingly, the cell population in the S phase is reduced relative to the control group. This effect is more pronounced in the sample with a thinner silica shell. In the case of the sample UCNP@thin_NH_2_, the percentage of the cell population in the second rest phase (G2) is strongly increased, whereas for the sample UCNP@thick_NH_2_ this percentage is comparable to that of the control. The calculation of the proliferative index (PI), according to Equation 1, shows that cells treated with both types of nanoparticles show a significantly decreased PI value (0.39 ± 0.05 for the UCNP@thin_NH_2_ sample and 0.35 ± 0.14 for the UCNP@thick_NH_2_ sample) compared to the control (0.53 ± 0.06).

[1] $$\text{PI}=\frac{\text{S}+\text{G2}/\text{M}}{\text{G}0/\text{G}1+\text{S}+\text{G2}/\text{M}}$$

In contrast to this observation, the silica particles without a UCNP core (sample NP@SiO_2_-RBITC-NH_2_) exhibit similar cell-cycle dynamics as the nanoparticle-free control (Figure S4, Supporting Information File 1). Their PI value (0.54 ± 0.10) is also similar to that of the control (0.48 ± 0.06) and of the control used for the other particles (0.53 ± 0.06).

The observation that an affected cell cycle has longer rest phases and a shorter S phase, especially for cells exposed to particles with a thinner silica shell, roughly correlates with the reduced cell viability of these samples in MTT assays. However, it is surprising that the cell cycle of cells treated with UCNP-free silica particles is not significantly influenced. Similar findings suggesting a partial blocking of the cell cycle by UCNPs were reported by Liu et al., who also observed a G0/G1 cell-cycle arrest and a significant decrease of the PI for human gastric adenocarcinoma (SGC-7901) cells incubated with poly(*N*-vinylpyrrolidone) (PVP)-coated NaYF_4_:Yb,Er particles in a similar concentration range [74]. Chen et al. investigated NaYF_4_:Yb,Er nanoparticles capped with (aminomethyl)phosphonic (AMPA), (aminopropyl)triethoxysilane (APTES), and dihydrocinnamic acid (DHCA) on epithelial cells (Chinese hamster ovary cells, CHO-K1) [75]. They observed that the positively charged AMPA and APTES UCNPs, as well as the negatively charged DHCA-capped particles led to a severe dysregulation of the cell cycle. In contrast to the present results, the authors found a dramatic decrease in the proportion of cells in the G1 phase and a substantial increase in the proportion of cells in the G2 phase. The latter is in line with an increase in dead or lysed cells compared to the untreated control. According to Chen et al., the amino-functionalized UCNPs were only capped at the surface with silane and no closed silica shell had grown around the particles, such that the release of cytotoxic ions was not reduced as in the present work.

Lu et al. investigated the effect of silica particles of various sizes and surface coatings on RAW.246.7 cells. Their results are in agreement with our findings, which demonstrate that amino-functionalized silica particles have only a negligible impact on the cell cycle given that they are in the same size and concentration range as in the present work [76]. These results suggest that silica is a suitable coating material to reduce cytotoxicity. The effect of UCNPs on RAW 246.7 cells has not been studied so far.

## Conclusion

In bioimaging applications using UCNPs, it is crucial to keep the particles intact (i.e., without dissolution processes) in the cellular environment as these processes reduce the viability of the investigated cells due to ion release. Coating NaYF_4_:Yb,Er nanocrystals with silica shells with two different thickness values is an efficient way to reduce the release of toxic ions from these particles and, consequently, their cytotoxicity. This assumption is well supported by cell viability, ion release, cellular uptake, and cell cycle assays, even if other factors (e.g., surface functionalization and subsequent effects, such as agglomeration) also influence these processes. However, it was shown that silica shells with thickness values of 7 nm or even 21 nm were not sufficient to completely hinder the release of lanthanide ions from UCNPs. According to MTT assays and, more specifically, cell cycle analysis, the UCNPs did not exhibit a biocompatibility level similar to that of silica particles without a lanthanide core. It has to be considered that amorphous silica obtained from a Stöber-like growth process is an inherently porous material with a pore size of 1–4 nm [35–36]. Thus, it contains pores that are larger than water molecules, as well as lanthanide and other ions (e.g., Na^+^ and F^−^) that are contained in UCNPs. An increase in the silica shell thickness likely reduces ion release. Moreover, ligands that actively reduce the release/dissolution process of lanthanide nanocrystals, such as (multi)chelating phosphonates [30,32–33], can be bound into or onto the surface of silica shells [77]. The use of different surfactants during the shell growth process might allow for a slightly further reduction of the pore size. Silica coating of UCNPs is a simple and well-established process. The thickness of the silica shell on UCNPs can easily be adjusted over a wide range up to 200 nm depending on the intended application [38]. Hence, it opens up not only a variety of possibilities for the (bio)-functionalization of UCNPs [52], but also it provides a simple approach to make UCNPs less cytotoxic.

## Experimental

All synthesis procedures were performed with standard glass equipment. Before use, the reaction vessels were cleaned with hydrofluoric acid (8 vol %) and repeatedly rinsed with water. The nanoparticles were redispersed using an ultrasonic bath operating at 860 W, 35 kHz (Sonorex RK512H, Bandelin). Ultrapure water (filter size = 0.22 μm, *R* = 18.2 MΩ·cm, Millipore) was used for all synthesis procedures.

### Materials

Oleic acid (OA, 90%), erbium chloride hexahydrate (ErCl_3_·6H_2_O, 99.9%), ytterbium chloride hexahydrate (YbCl_3_·6H_2_O, 99.9%), and yttrium chloride hexahydrate (YCl_3_·6H_2_O, 99.9%) were purchased from ABCR. *N*-(6-Aminohexyl)-3-aminopropyltrimethoxysilane (AHAPS, 97%), 3-aminopropyltrimethoxysilane (APS, 99%), rhodamine B isothiocyanate (RBITC, ≥95%), polyoxyethylene-(5)-nonylphenyl ether (Igepal^®^ CO-520), ammonium fluoride (NH_4_F, 99.8%), 1-octadecene (tech. 95%), sodium oleate (82%), tetraethyl orthosilicate (TEOS, 98%), as well as erbium, yttrium, and ytterbium standards for ICP-OES measurements (TraceCERT^®^, *c* = 1000 mg/mL) were obtained from Sigma-Aldrich. Cyclohexane (tech. 99.5%) and ammonia water (p.a., 25 wt % NH_3_) were purchased from Roth. Ethanol (EtOH, 100%) was purchased from Berkel AHK, hydrofluoric acid (HF, 30%) was purchased from Riedel de Haën, and sodium hydroxide (NaOH, 99%) was purchased from Grüssing.

DMEM, FBS, antibiotics, and PBS (pH 7.4) were purchased from Life Technologies (Carlsbad, CA, USA). 3-(4,5-Dimethylthiazol-2-yl)-2,5-diphenyltetrazolium bromide (MTT), dimethyl sulfoxide (DMSO), propidium iodide, and RNase were obtained from Sigma-Aldrich (St. Louis, MO, USA). The T-75 and T-25 flasks, used for growing the cells, and the 12- and 96-well plates were purchased from Corning^®^. Cell scrapers used to scrape RAW 264.7 cells from the bottom of the flasks were purchased from PLC Labclinics. All chemicals were used without further purification.

### Synthesis

NaYF_4_:Yb,Er UCNPs were synthesized from the corresponding lanthanide oleates [78–79] according to a modified procedure from Na et al. [47], which is described in detail in [38].

#### Growth of silica shells

For silica shells (thickness = 7 ± 1 nm), a dispersion of UCNPs (diameter = 33 ± 2 nm; *c* = 3 g/L) in 33.3 mL of cyclohexane was used. After sonicating for 10 min, 3.736 mL of Igepal CO-520 was added. After a brief mixing using an ultrasonic bath, 0.331 mL of ammonia water was added, and the dispersion was sonicated again for 20 min. Subsequently, 0.331 mL of TEOS was added and the whole mixture was sonicated for at least 1 h. Finally, the dispersion was stirred for 12 h at 1200 rpm at room temperature [38].

For growing 21 ± 2 nm thick silica shells, additional cyclohexane, Igepal CO-520, and ammonia water were added to the non-purified dispersion of UCNPs coated with 7 ± 1 nm thick shells to maintain a surfactant concentration of 11 wt % and a maximum water concentration of 2–3 wt %. The initial concentration of UCNP cores was set to 20 g/L and the total volume was 5 mL. Next, 1.551 mL of TEOS was added stepwise at a rate of 20.8 μL/min through a peristaltic pump (REGLO Digital MS–2/8–160, Ismatec, with a TYGON R-3603 tubing, type AME-01) while the dispersion was stirred for 12 h at 1200 rpm at room temperature. When the desired shell thickness was reached, the particles were precipitated by adding 5–10 mL of EtOH, purified by three cycles of centrifugation (1200*g*, 1 h) and redispersion in 10 mL of EtOH. Finally, the particles were redispersed in 10–15 mL of EtOH [38].

For the growth of silica shells with covalently bound RBITC, a modified method from Verhaegh et al. was used [80]. The reaction was carried out under inert atmosphere. The dye was first coupled with APS yielding the dye-coupling product RBITC-APS. For this, 2.7 mg (5 ± 10^−3^ mmol) of RBITC was diluted to 1 mM in absolute EtOH and 10 µL (5 ± 10^−2^ mmol) of APS was added. The solution was stirred overnight at room temperature under inert atmosphere and the coupling product was not purified. The growth of the silica shell was performed as described above. In addition, ammonia water was added as the last reagent and, after the addition of TEOS, 108 µL of the ethanolic solution of RBITC-APS was continuously added dropwise through a syringe in the case of the particles with thin silica shells for 100 mg mass of non-coated UCNPs. In the case of the second growth step of the thicker shells, 432 µL of this solution was added instead.

To obtain a positive surface charge, silica-coated UCNPs were functionalized with AHAPS. The reaction was carried under inert atmosphere and it was modified from [51]. As an example, in the case of the particles with thin shells, 1.5 mL (*c* = 20 g/L in ethanol, particle mass = 30 mg) of the nanoparticle dispersion was diluted in ethanol to *c* = 1 g/L. To ensure that the entire surface was covered with AHAPS and to keep the pH value at 9, a ten-time excess of 30 µL of AHAPS and a few drops of ammonia water (30% v/v) were added, respectively. The mixture was stirred overnight under argon atmosphere, followed by heating under reflux for 1 h. For the particles with thicker silica coating, 1.5 mL (*c* = 20 g/L in ethanol, particle mass = 30 mg) of the dispersion was diluted to *c* = 1 g/L and reacted with 25 µL of a solution containing AHAPS and ammonium hydroxide. The nanoparticles were washed three times under inert atmosphere by repeated centrifugation (1200*g*, 1 h) and redispersion in 10 mL of EtOH. Finally, the particles were redispersed in 10–15 mL of EtOH.

The silica particles without an UCNP core were prepared as described in [51]. However, instead of fluorescein isothiocyanate, rhodamine isothiocyanate was used. The functionalization with AHAPs was carried out by using the same procedure, which was also used for the silica-coated UCNPs.

### Characterization

#### Scanning transmission electron microscopy

STEM images were taken using a Hitachi SU 8030 scanning electron microscope with an electron acceleration voltage of 30 kV and a current of 20 µA. A droplet of the nanoparticle dispersion (*c* = 0.5–1 g/L) in either cyclohexane, for oleate-functionalized UCNP cores, or in ethanol, for silica-coated UCNPs, was dried on a carbon-coated copper grid (Cu 400 mesh, Quantifoil^®^: 100 carbon support films). The images were analyzed using the software FIJI. At least 300 particles per sample were analyzed.

#### Dynamic light scattering and electrophoretic light scattering

The DLS and ELS measurements were performed using a Zetasizer Nano ZS system (Malvern Instruments) at 25 °C and at a wavelength of 633 nm. The uncoated UCNPs were dispersed in cyclohexane, whereas the silica-coated particles were dispersed in ethanol, water, or in supplemented DMEM. Then, they were filtered through sterile syringe filters (pore size: 0.2 µm, Rotilab). Nylon filters were used for particles dispersed in cyclohexane and ethanol, whereas regenerated cellulose filters were used for particles dispersed in water or DMEM. Zeta potential measurements of the dispersions in ethanol and water were carried out using capillary zeta cells (DTS 1070, Malvern Instruments). In all measurements, the concentration of the samples was in the range of 0.5–1 mg/mL.

#### Ion release experiments

The silica-coated UCNPs were redispersed in 4 mL of ultrapure water or supplemented DMEM, such that a final particle concentration of 200 μg/mL for the silica-coated UCNPs and for the UCNP cores was obtained. The dispersions were kept at 37 °C for 24 h, and centrifuged with centrifuge tubes containing membrane filters (Amicon ultracentrifuge, low-binding ultracel membrane, 3000 molecular weight cutoff (MWCO)) for 2 h at 3080*g*. Aliquots were diluted in 10 mL of a solution containing ultrapure water and aqua regia (water/aqua regia = 4:1 v/v) and measured via ICP-OES for determining the concentration of the Er^3+^, Yb^3+^, and Y^3+^ ions (Supporting Information File 1).

Aqueous solutions containing 1.0 ± 0.1 and 2.0 ± 0.1 ppm of Er^3+^, Yb^3+^, or Y^3+^ (prepared from the corresponding lanthanoid chlorides) were also centrifuged through the same Amicon filter tubes mentioned above, diluted in 10 mL of ultrapure water and aqua regia (water/aqua regia = 4:1 v/v) solution, and analyzed via ICP-OES to determine the concentration of the Er^3+^, Yb^3+^, or Y^3+^ ions (Supporting Information File 1).

### Cell culture of RAW 264.7 cells

RAW 264.7 cells were provided by the group of Dr. Philipp Seib at the University of Strathclyde, Glasgow, UK. The cells were grown in DMEM medium supplemented with 10% FBS, 2 mM L-glutamine, 100 U/mL penicillin, 100 μg/mL streptomycin, and 250 μg/mL fungizone at 37 °C in a 5% CO_2_ humidified atmosphere [73]. The cells were observed daily for confluence and cell morphology by using an inverted phase-contrast Eclipse TS100 microscope (Nikon, Tokyo, Japan). For routine subculturing, cells at approx. 80% confluency were gently lifted off by scrapping and transferred into fresh growth medium. For each experiment, cells were allowed to adhere for 24 h, and then the medium was replaced with fresh medium containing UCNPs.

### MTT cell viability assay

Cell viability was determined by the colorimetric changes in the MTT cytotoxicity assay. For that, 10^3^ cells per well were seeded into a 96-well Corning plate. Cells were, then, incubated for 24 h at 37 °C in a 5% CO_2_ humidified atmosphere. After that, the culture medium was replaced with fresh medium containing UCNPs at 12.5, 25, 50, 100, 150, and 200 µg/mL for 24 h. RAW 264.7 cells exposed to culture medium without UCNPs were used as controls. Then, 50 µL of MTT at 1 mg/mL in PBS was added to each well and the cells were incubated for another 4 h at 37 °C in 5% CO_2_. Afterward, 150 µL of DMSO was added to each well and the plates were shaken in the dark using an orbital shaker (Mini Shaker, Kisker Biotec). The absorbance was recorded at 570 nm using a microtiter plate reader (Synergy HT, BioTeK Instruments Inc).

The percentage of cell growth inhibition was calculated using Equation 2:

[2] $$\text{\% of inhibition}=\frac{\begin{array}{l} \text{absorption at 570 nm from}\\ \text{sample} \end{array}}{\begin{array}{l} \text{absorption at 570 nm from}\\ \text{negative control} \end{array}}\cdot 100\%$$

### Cell cycle analysis

The cell cycle was analyzed by using flow cytometry, according to the method previously described [81]. Briefly, cells were seeded onto 6-well plates and incubated with UCNPs at a concentration of 200 µg/mL. After exposure, the cells were washed with PBS, harvested through scrapping, and centrifuged twice at 300*g* for 5 min. Cells were then fixed with 85% ice-cold ethanol and kept at −20 °C until analysis. At the time of analysis, cells were centrifuged at 300*g* for 5 min, resuspended in PBS, and filtered through a 50 µm nylon mesh to separate aggregates. Cells were then incubated with 50 µL of propidium iodide (1 mg/mL), a DNA intercalating fluorochrome, and 50 µL of RNase (1 mg/mL) for 20 min, in the dark and at room temperature. Cell cycle distribution data was assessed by using a Beckman Coulter EPICS XL flow cytometer (Coulter Electronics, Hialeah, Florida, USA), and the percentage of cells in sub-G1, G0/G1, S, and G2 phases was determined by using the FlowJo software (FlowJo LLC, Ashland, OR, USA) applying the Watson Pragmatic model.

### Uptake potential analysis by flow cytometry

The uptake potential of UCNPs by RAW 264.7 cells was obtained by using flow cytometry. RAW 264.7 cells were seeded (10^5^ per well) onto a 12-well plate and incubated for 24 h at 37 °C in a 5% CO_2_ humidified atmosphere in order to adhere to the bottom of the wells. After that, the medium was replaced with fresh medium containing nanoparticles at a concentration of 100 µg/mL. Fresh medium, without particles, was added to the control and blank wells. Cells were incubated for 24 h at 37 °C. After that, the supernatant was removed from each well, and the cells were washed once with PBS. Then, 1 mL of supplemented DMEM was added and the cells were finally collected by scraping and analyzed via flow cytometry in an Attune^®^ Acoustic Focusing Cytometer (ThermoFisher Scientific). Both forward-scatter (FS), which provides information regarding particle size, and side-scatter (SS), which provides information regarding the complexity of the particles, parameters were measured.

## Supporting Information

File 1 Experimental details, additional UC luminescence spectra, XRD data, STEM images, ICP-OES, and cell cycle data.

## References

[1] Lu Y, Zhao J, Zhang R, Liu Y, Liu D, Goldys E M, Yang X, Xi P, Sunna A, Lu J (2014). Nat Photonics.

[2] Bettinelli M, Carlos L, Liu X (2015). Phys Today.

[3] Sun S-K, Wang H-F, Yan X-P (2018). Acc Chem Res.

[4] Li Z, Yuan H, Yuan W, Su Q, Li F (2018). Coord Chem Rev.

[5] Li X, Zhang F, Zhao D (2015). Chem Soc Rev.

[6] Jalani G, Tam V, Vetrone F, Cerruti M (2018). J Am Chem Soc.

[7] Zhou M, Ge X, Ke D-M, Tang H, Zhang J-Z, Calvaresi M, Gao B, Sun L, Su Q, Wang H (2019). Front Chem (Lausanne, Switz).

[8] Dukhno O, Przybilla F, Muhr V, Buchner M, Hirsch T, Mély Y (2018). Nanoscale.

[9] Oliveira H, Bednarkiewicz A, Falk A, Fröhlich E, Lisjak D, Prina-Mello A, Resch S, Schimpel C, Vrček I V, Wysokińska E (2019). Adv Healthcare Mater.

[10] Plohl O, Kraft M, Kovač J, Belec B, Ponikvar-Svet M, Würth C, Lisjak D, Resch-Genger U (2017). Langmuir.

[11] Kostiv U, Rajsiglová L, Luptáková D, Pluháček T, Vannucci L, Havlíček V, Engstová H, Jirák D, Šlouf M, Makovicky P (2017). RSC Adv.

[12] Zhou L, Wang R, Yao C, Li X, Wang C, Zhang X, Xu C, Zeng A, Zhao D, Zhang F (2015). Nat Commun.

[13] Yang D, Ma P, Hou Z, Cheng Z, Li C, Lin J (2015). Chem Soc Rev.

[14] Tsai Y-C, Vijayaraghavan P, Chiang W-H, Chen H-H, Liu T-I, Shen M-Y, Omoto A, Kamimura M, Soga K, Chiu H-C (2018). Theranostics.

[15] Su Q, Feng W, Yang D, Li F (2017). Acc Chem Res.

[16] Chen B, Su Q, Kong W, Wang Y, Shi P, Wang F (2018). J Mater Chem B.

[17] Green K, Huang K, Pan H, Han G, Lim S F (2018). Front Chem (Lausanne, Switz).

[18] Palo E, Salomäki M, Lastusaari M (2019). J Colloid Interface Sci.

[19] Lahtinen S, Lyytikäinen A, Päkkilä H, Hömppi E, Perälä N, Lastusaari M, Soukka T (2017). J Phys Chem C.

[20] Lisjak D, Plohl O, Vidmar J, Majaron B, Ponikvar-Svet M (2016). Langmuir.

[21] Plohl O, Kralj S, Majaron B, Fröhlich E, Ponikvar-Svet M, Makovec D, Lisjak D (2017). Dalton Trans.

[22] Estebanez N, González-Béjar M, Pérez-Prieto J (2019). ACS Omega.

[23] Lisjak D, Plohl O, Ponikvar-Svet M, Majaron B (2015). RSC Adv.

[24] Mandl G A, Cooper D R, Hirsch T, Seuntjens J, Capobianco J A (2019). Methods Appl Fluoresc.

[25] Agalakova N I, Gusev G P (2012). ISRN Cell Biol.

[26] Barbier O, Arreola-Mendoza L, Del Razo L M (2010). Chem-Biol Interact.

[27] Pałasz A, Czekaj P (2000). Acta Biochim Pol.

[28] Gnach A, Lipinski T, Bednarkiewicz A, Rybka J, Capobianco J A (2015). Chem Soc Rev.

[29] Elsaesser A, Howard C V (2012). Adv Drug Delivery Rev.

[30] Li R, Ji Z, Dong J, Chang C H, Wang X, Sun B, Wang M, Liao Y-P, Zink J I, Nel A E (2015). ACS Nano.

[31] Liu J-N, Bu W-B, Shi J-L (2015). Acc Chem Res.

[32] Kostiv U, Lobaz V, Kučka J, Švec P, Sedláček O, Hrubý M, Janoušková O, Francová P, Kolářová V, Šefc L (2017). Nanoscale.

[33] Li R, Ji Z, Chang C H, Dunphy D R, Cai X, Meng H, Zhang H, Sun B, Wang X, Dong J (2014). ACS Nano.

[34] Hemmer E, Yamano T, Kishimoto H, Venkatachalam N, Hyodo H, Soga K (2013). Acta Biomater.

[35] Li S, Wan Q, Qin Z, Fu Y, Gu Y (2015). Langmuir.

[36] Raschpichler C, Goroncy C, Langer B, Antonsson E, Wassermann B, Graf C, Klack P, Lischke T, Rühl E (2020). J Phys Chem C.

[37] Plohl O, Majaron B, Ponikvar-Svet M, Makovec D, Lisjak D (2015). Acta Chim Slov.

[38] Kembuan C, Saleh M, Rühle B, Resch-Genger U, Graf C (2019). Beilstein J Nanotechnol.

[39] Wang K, Ma J, He M, Gao G, Xu H, Sang J, Wang Y, Zhao B, Cui D (2013). Theranostics.

[40] Appelqvist H, Wäster P, Kågedal K, Öllinger K (2013). J Mol Cell Biol.

[41] Iler R K (1979). The Chemistry of Silica: Solubility, Polymerization, Colloid and Surface Properties and Biochemistry of Silica.

[42] Wysokińska E, Cichos J, Kowalczyk A, Karbowiak M, Strządała L, Bednarkiewicz A, Kałas W (2019). Biomolecules.

[43] Dussert F, Arthaud P-A, Arnal M-E, Dalzon B, Torres A, Douki T, Herlin N, Rabilloud T, Carriere M (2020). Nanomaterials.

[44] Dalzon B, Aude-Garcia C, Collin-Faure V, Diemer H, Béal D, Dussert F, Fenel D, Schoehn G, Cianférani S, Carrière M (2017). Nanoscale.

[45] Kurtz-Chalot A, Villiers C, Pourchez J, Boudard D, Martini M, Marche P N, Cottier M, Forest V (2017). Mater Sci Eng, C.

[46] Nabeshi H, Yoshikawa T, Arimori A, Yoshida T, Tochigi S, Hirai T, Akase T, Nagano K, Abe Y, Kamada H (2011). Nanoscale Res Lett.

[47] Na H, Woo K, Lim K, Jang H S (2013). Nanoscale.

[48] Chen G, Qiu H, Prasad P N, Chen X (2014). Chem Rev.

[49] Haase M, Schäfer H (2011). Angew Chem, Int Ed.

[50] Fujii M, Nakano T, Imakita K, Hayashi S (2013). J Phys Chem C.

[51] Graf C, Gao Q, Schütz I, Noufele C N, Ruan W, Posselt U, Korotianskiy E, Nordmeyer D, Rancan F, Hadam S (2012). Langmuir.

[52] Zhang J, Liu F, Li T, He X, Wang Z (2015). RSC Adv.

[53] Wang Y, Zhao Q, Han N, Bai L, Li J, Liu J, Che E, Hu L, Zhang Q, Jiang T (2015). Nanomedicine (N Y, NY, U S).

[54] Izak-Nau E, Voetz M, Eiden S, Duschl A, Puntes V F (2013). Part Fibre Toxicol.

[55] Monopoli M P, Walczyk D, Campbell A, Elia G, Lynch I, Baldelli Bombelli F, Dawson K A (2011). J Am Chem Soc.

[56] Pisani C, Rascol E, Dorandeu C, Gaillard J-C, Charnay C, Guari Y, Chopineau J, Armengaud J, Devoisselle J-M, Prat O (2017). PLoS One.

[57] Saleh M I, Rühle B, Wang S, Radnik J, You Y, Resch-Genger U (2020). Sci Rep.

[58] Qiu Y, Liu Y, Wang L, Xu L, Bai R, Ji Y, Wu X, Zhao Y, Li Y, Chen C (2010). Biomaterials.

[59] Lundqvist M, Stigler J, Elia G, Lynch I, Cedervall T, Dawson K A (2008). Proc Natl Acad Sci U S A.

[60] Lettinga M P, van Zandvoort M A M J, van Kats C M, Philipse A P (2000). Langmuir.

[61] Dong C, Pichaandi J, Regier T, van Veggel F C J M (2011). J Phys Chem C.

[62] Itoh H, Hachiya H, Tsuchiya M, Suzuki Y, Asano Y (1984). Bull Chem Soc Jpn.

[63] Wu D, Wu X, Lv Y, Wang H (2008). Mater Lett.

[64] Xia T, Kovochich M, Liong M, Zink J I, Nel A E (2008). ACS Nano.

[65] Liu Z, Escudero A, Carrillo-Carrion C, Chakraborty I, Zhu D, Gallego M, Parak W J, Feliu N (2020). Chem Mater.

[66] Jochums A, Friehs E, Sambale F, Lavrentieva A, Bahnemann D, Scheper T (2017). Toxics.

[67] Suzuki H, Toyooka T, Ibuki Y (2007). Environ Sci Technol.

[68] Zucker R M, Daniel K M, Massaro E J, Karafas S J, Degn L L, Boyes W K (2013). Cytometry, Part A.

[69] Kumar A, Pandey A K, Singh S S, Shanker R, Dhawan A (2011). Cytometry, Part A.

[70] Zucker R M, Massaro E J, Sanders K M, Degn L L, Boyes W K (2010). Cytometry, Part A.

[71] Toduka Y, Toyooka T, Ibuki Y (2012). Environ Sci Technol.

[72] Rosário F, Hoet P, Santos C, Oliveira H (2016). Toxicology.

[73] Bastos V, Ferreira de Oliveira J M P, Brown D, Jonhston H, Malheiro E, Daniel-da-Silva A L, Duarte I F, Santos C, Oliveira H (2016). Toxicol Lett.

[74] Liu C, Chen H, Li S, Xu H, Zhao D (2016). J Nanosci Nanotechnol.

[75] Chen Y, D'Amario C, Gee A, Duong H T T, Shimoni O, Valenzuela S M (2020). Acta Biomater.

[76] Lu X, Jin T, Zheng J, Fan X (2017). J Nanosci Nanotechnol.

[77] Duncan A K, Klemm P J, Raymond K N, Landry C C (2012). J Am Chem Soc.

[78] Park J, An K, Hwang Y, Park J-G, Noh H-J, Kim J-Y, Park J-H, Hwang N-M, Hyeon T (2004). Nat Mater.

[79] Wei Y, Lu F, Zhang X, Chen D (2006). Chem Mater.

[80] Verhaegh N A M, van Blaaderen A (1994). Langmuir.

[81] Oliveira H, Monteiro C, Pinho F, Pinho S, Ferreira de Oliveira J M P, Santos C (2014). Mutat Res, Genet Toxicol Environ Mutagen.

