# Supplementary material for: Effect of different silica coatings on the toxicity of upconversion nanoparticles on RAW 264.7 macrophage cells
Source: Beilstein J Nanotechnol. 2021 Jan 8;12:35–48. doi: 10.3762/bjnano.12.3 (PMC7801781; doi:10.3762/bjnano.12.3)
Supplement: File 1 — Experimental details, additional UC luminescence spectra, XRD data, STEM images, ICP-OES, and cell cycle data. [file Beilstein_J_Nanotechnol-12-35-s001.pdf]

BEILSTEIN JOURNAL OF NANOTECHNOLOGY

Supporting Information

for

Effect of different silica coatings on the toxicity of upconversion nanoparticles on RAW 264.7 macrophage cells

Cynthia Kembuan, Helena Oliveira and Christina Graf

Beilstein J. Nanotechnol. **2021,** *12,* 35–48. doi:10.3762/bjnano.12.3

Experimental details, additional UC luminescence spectra, XRD data, STEM images, ICP-OES, and cell cycle data

License and Terms: This is a supporting information file under the terms of the Creative Commons Attribution License (https://creativecommons.org/licenses/by/4.0). Please note that the reuse, redistribution and reproduction in particular requires that the author(s) and source are credited and that individual graphics may be subject to special legal provisions.
The license is subject to the *Beilstein Journal of Nanotechnology* terms and conditions: (https://www.beilstein-journals.org/bjnano/terms)

X-ray diffraction (XRD)

For XRD measurements, a minimum amount of 10 mg dried samples were used. The XRD device was a STOE Stadi P from STOE. A Cu $K_{\alpha 1}$ radiation source with a radiation wavelength of 0.15405 nm was used. The measurement angle was between 10-90° and with a measurement time of 120 s/0.2°.

Measurements of the upconversion luminescence

The upconversion luminescence (UCL) was measured at 25°C with a *FluoroMax-4* spectrometer from *Horiba Jobin Yvon* equipped with a 2 W 980 nm laser diode from *Insaneware-Robert Nowak*. The concentration of the samples was 1-2 g/L in cyclohexane for oleate-capped UCNPs or ethanol for silica-coated UCNPs, and quartz glass cuvettes (*QS Suprasil*, 5 mm, *Hellma* or *VWR)* were used.

Inductively coupled plasma-optical emission spectroscopy (ICP-OES)

The elemental composition of the UCNP cores was determined by ICP-OES. For this purpose, 1 mL (c = 5 g/L) of their dispersion in cyclohexane was dried. The dried UCNPs were subsequently dissolved in 1 mL of aqua regia for 30 minutes and diluted with 5 mL of ultrapure water. The measurements were performed with an iCAP 6000 Series ICP Spectrometer from Thermo Scientific with a radial optical approach. For calibration, series of solutions with different concentrations were prepared separately from an erbium standard for ICP ($c(Er^{3+})$ = 1, 5, 10 ppm), ytterbium standard for ICP ($c(Yb^{3+})$ = 10, 20, 40 ppm), and an yttrium standard for ICP ($c(Y^{3+})$ = 10, 20 and 40 ppm.

X-ray diffraction measurements

The XRD diffractogram (Figure S1) shows a predominantly hexagonal crystal structure for example at 18°, 29°, 44° and 54° (ICDD no. 28-1192), with two minor peaks from the α-phase at 47° for [220] reflex and 55° for [311] reflex (ICDD no. 06-0334; see Figure S1).

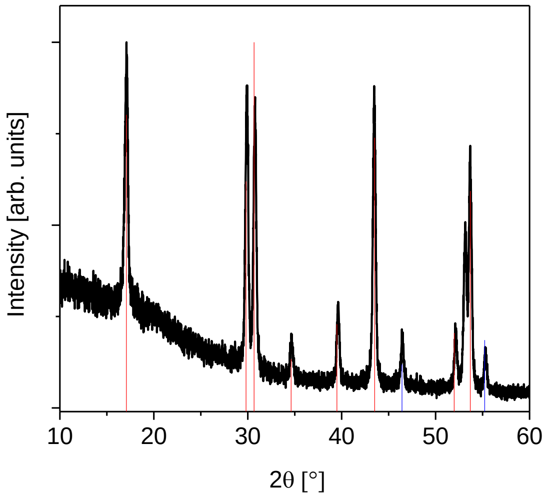

Figure S1: XRD diffractogram of the $NaYF_4$: Yb, Er cores (red lines: hexagonal phase peaks (ICDD no. 28-1192); blue lines: cubic phase peaks (ICDD no. 06-0334).

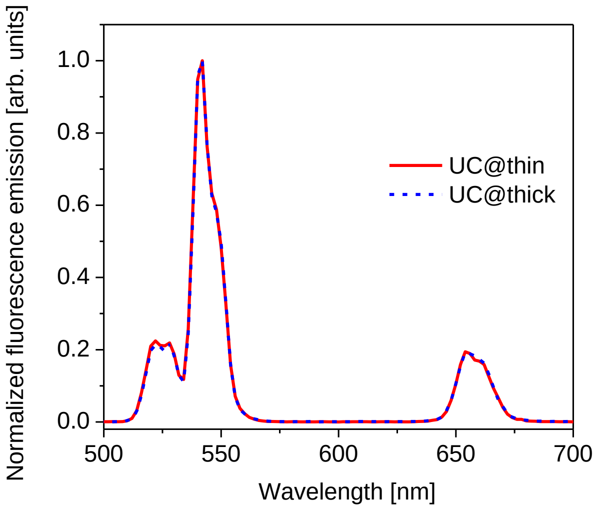

Figure S2: Upconversion luminescence spectra of UC@thin_NH_2 (r_{SiO2} = 8 ± 2 nm) and UC@thick_NH_2 (r_{SiO2} = 21 ± 2 nm) in ethanol. The cores of both particles are $NaYF_4$: 18% Yb, 2% Er nanoparticles. The spectra are normalized at 655 nm for better comparison. The excitation power density was 2 W/cm^2 at 980 nm.

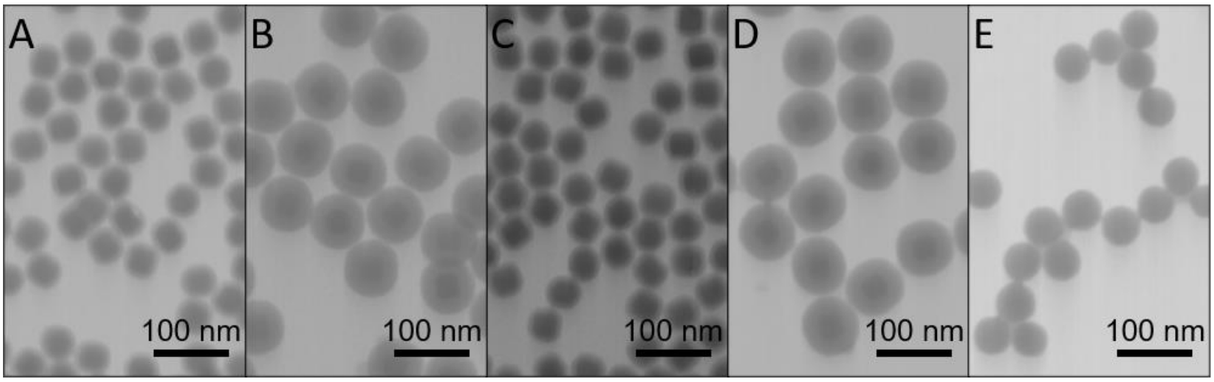

Figure S3: STEM images of A: UC@thin_NH_2 (r_{SiO2} = 8±2 nm); B: UC@thick_NH_2 (r_{SiO2} = 21±2 nm; C: UC@thin_RBITC_NH_2 (r_{SiO2} = 9±2 nm); D: UC@thick_RBITC_NH_2 (r_{SiO2} = 22±2 nm) and E: functionalized SiO_2-nanoparticles SiO_2@RBITC_NH_2 (average STEM-diameter = 52±3 nm). The cores of all particles are $NaYF_4$: 18% Yb, 2% Er nanoparticles

Table S1: Filtered lanthanide ions value from the corresponding chlorides obtained from ICP-OES measurement.

Initial ions concentration	Y		Yb		Er	
Concentration [ppm]	Concentration [mmol/L]	Ions filtered [%]	Concentration [mmol/L]	Ions filtered [%]	Concentration [mmol/L]	Ions filtered [%]
1	$[4.30 \pm 0.05]\cdot10^{-3}$	38±3	$[1.00 \pm 0.01]\cdot10^{-3}$	17 ± 2	$[3.32 \pm 0.05]\cdot10^{-4}$	6.0 ± 0.6
2	$[1.00 \pm 0.01]\cdot10^{-2}$	45±4	$[2.40 \pm 0.01]\cdot10^{-3}$	21 ± 2	$[7.48 \pm 0.05]\cdot10^{-4}$	6.4 ± 0.6

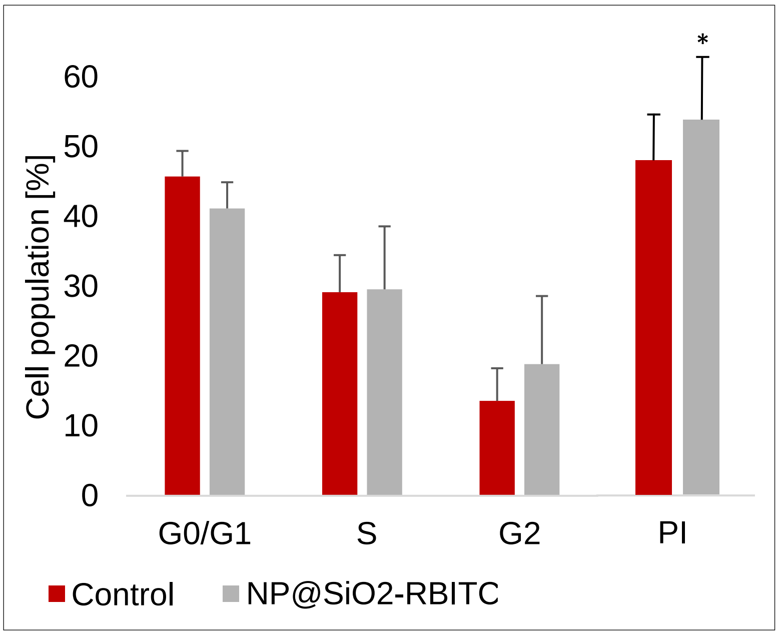

Figure S4: Effect of silica particles without a UCNP core (NP@SiO_2-RBITC-NH_2) on the cell cycle dynamics of RAW 264.7 macrophages after 24 h of exposure. The concentration was 200 µg/mL.
